# Developing a digital interactive tool to enhance cervical cancer awareness and health literacy in Croatia: a community case study

**DOI:** 10.3389/fpubh.2026.1767197

**Published:** 2026-04-13

**Authors:** Tatjana Nemeth Blažić, Qi Zhu, Elena Cahun, Iskra Alexandra Nola

**Affiliations:** 1 Department for HIV, Sexual and Blood Transmitted Diseases, Croatian Institute of Public Health, Zagreb, Croatia; 2 Polyclinic Poliderma, Zagreb, Croatia; 3 Primary Health Care Center Zagreb – Centre, Zagreb, Croatia; 4 Andrija Štampar School of Public Health, School of Medicine, University of Zagreb, Zagreb, Croatia

**Keywords:** cervical cancer, Croatia, digital interactive tool, health education, health literacy, self-assessment

## Abstract

**Background:**

Cervical cancer is highly preventable but remains a major public health problem due to limited health literacy and suboptimal uptake of HPV vaccination and screening. Digital health tools are increasingly used to provide risk communication and prevention information.

**Objective:**

To describe the development, implementation, and early use of a free web-based tool for cervical cancer risk self-assessment in Croatia and to report initial usage metrics from this community-based initiative.

**Methods:**

An interdisciplinary team translated published evidence on cervical cancer risk factors into a decision-tree-based scoring system embedded in an online questionnaire featuring conditional logic and tailored feedback. Risk categories and thresholds were derived from literature-informed estimates and expert consensus and have not yet been empirically validated against clinical outcomes. The tool was deployed on the national “Neću rak” (“I Don’t Want Cancer”) platform of the Croatian Institute of Public Health. Aggregated, anonymized usage data from February 2023 to February 2025 were analysed descriptively.

**Results:**

The questionnaire (10–14 items, depending on branching logic) categorises users into four qualitative risk levels (no, low, moderate, high). In the first two years, the tool was accessed almost 9,000 times, with about 8,000 completed questionnaires, indicating a high completion rate. Most respondents (around 60%) were aged 34–54 years. Self-reported HPV vaccination (13%) and smoking history (30%) reflect aggregated responses from voluntary users and do not represent the general population.

**Conclusion:**

This national web-based risk calculator demonstrates the feasibility of integrating evidence-informed scoring framework and tailored educational content within a public health platform. Such digital tools may contribute to awareness and support existing screening and vaccination programmes. However, formal evaluation of algorithm performance, usability, and potential behavioural impact is required before effectiveness can be determined.

## 1 Introduction

Cervical cancer is one of the most preventable types of cancer. Despite the availability of efficient and cost-effective primary and secondary prevention strategies, it remains a major public health challenge in women’s health. According to World Health Organization (WHO) data, cervical cancer is the fourth most common cancer in women globally, with around 660,000 new cases and approximately 350,000 deaths annually, accounting for nearly 8% of all female cancer deaths each year (1, 2). Although the disease is serious, it can be effectively prevented and is almost completely curable if detected early or in a premalignant stage.

Two key preventive measures: human papillomavirus (HPV) vaccination and organised cervical cancer screening, have demonstrated substantial impact on incidence and mortality when implemented at scale. However, successful implementation depends not only on the availability of these services but also on women’s health literacy, awareness of risk and protective factors, and willingness to engage in preventive behaviours. Low levels of knowledge about HPV and the HPV vaccine are among the most common reasons for refusing vaccination, not recommending the vaccine to peers, and negative parental decision-making regarding HPV vaccination (3, 4). Poor health literacy is likewise a major reason for delayed, irregular, or absent screening among women in the target age range (5).

The WHO, in its strategies and action plans, emphasises digital health as a key enabler for health promotion, disease prevention and patient engagement, including for cervical cancer prevention (6, 7). Digital tools can lower access barriers, offer tailored information at scale and support shared decision-making. Within this context, national and local public health institutions are increasingly exploring digital interventions that combine risk communication, self-assessment, and education.

As part of the educational and informative activities of the Croatian National Programme for Early Detection of Cervical Cancer, the Croatian Institute of Public Health developed a free, publicly available web-based digital tool—hereafter referred to as the Cervical Cancer Risk Calculator (C2RC). The tool aims to increase health literacy about risk and protective factors and to provide a rough risk stratification for developing cervical cancer, framed explicitly as an educational and awareness-raising instrument rather than a diagnostic tool.

This community case study describes the context, development, and implementation of the C2RC and presents early usage statistics from its first two years online. We also discuss lessons learned and conceptual and methodological constraints to inform others planning similar digital public health interventions.

## 2 Context

### 2.1 National epidemiology and prevention programmes

Cervical cancer remains an important public health concern in Croatia, with incidence and mortality rates comparable to those of other Central and Eastern European countries. Approximately 300 new cases of cervical cancer and around 120 related deaths are recorded annually. Unlike many other cancers, cervical cancer disproportionately affects younger women; in 2021, one-third of newly diagnosed cases occurred in women under 50 years of age, and it ranked as the third most common malignancy among women aged 30–39. Mortality remains a concern, with 121 deaths reported in 2023 (6.1 per 100,000), and approximately one-third of deaths occurring in women younger than 60 years.

Organised cervical cancer screening and introduction of HPV vaccination into national immunisation schedule have expanded preventive opportunities; however, coverage of both vaccination and screening remains suboptimal and uneven across age groups, educational levels, and regions (36–38). Despite recent improvements in HPV vaccine uptake, full population-level coverage has not yet been achieved, and substantial inter-county variation persists.

The National Cervical Cancer Screening Programme has also faced implementation challenges. The first organised screening cycle (2012–2016) achieved a participation rate of 10.3%. Following temporary suspension due to infrastructural and technical constraints, the programme entered a reorganisation phase, with pilot implementation of revised protocols—including primary HPV testing—initiated in 2023 in one county. National roll-out remains contingent on evaluation of feasibility, organisational capacity, and resource availability.

These structural characteristics—moderate screening participation, regional disparities in vaccination coverage, and ongoing programme reorganisation—create a context in which accessible, standardised educational resources aligned with national prevention pathways may serve as supportive tools for informed participation in prevention and early detection efforts. Within the Croatian National Programme for Early Detection of Cervical Cancer, communication and educational activities are coordinated through the national “Neću rak” (“I Don’t Want Cancer”) digital platform, developed and maintained by the Croatian Institute of Public Health.

### 2.2 Digital and health literacy context

Internet and smartphone use and penetration in Croatia is high, particularly among young people, including women of reproductive age, making web-based interventions feasible as an adjunct to traditional health promotion channels. However, studies and routine programme data indicate persistent gaps in health literacy and limited awareness of HPV, cervical cancer risk factors and evidence-based prevention options (3–5, 13–15). These gaps are especially problematic because they influence both primary prevention (HPV vaccination, smoking cessation, safer sexual behaviour) and secondary prevention (participation in Pap testing or HPV-based screening).

Digital tools that allow users to explore their personal risk in a structured, guided way, while simultaneously providing explanations and links to trusted educational material, are a promising approach to address these gaps, particularly when embedded within a recognised public health institution’s platform.

### 2.3 Target population and setting

The C2RC is hosted on the “Neću rak” website of the Croatian Institute of Public Health and is free and openly accessible to the general population. The primary intended users are women with a cervix in the recommended age range for cervical cancer screening, particularly those who may be unsure about their personal risk or about the relevance of screening and vaccination. The tool estimates qualitative level of risk: high risk, moderate risk, low risk, no risk. It is also suitable for use by healthcare providers and counsellors as a conversation support instrument in primary care and gynaecological settings.

The national platform is promoted through media campaigns, social media channels and programme activities such as European Cancer Prevention Week and National Cervical Cancer Prevention Day (“Mimosa Day”), which typically generate increased traffic to the site.

## 3 Case description of key implementation elements: development of the cervical cancer risk calculator and usage data

### 3.1 Planning and algorithm development

The C2RC methodology is based on an interactive questionnaire supported by a rule-based scoring framework informed by established epidemiological evidence on cervical cancer risk factors (8–16). A comprehensive literature review identified key variables associated with cervical cancer risk, including sexual and reproductive behaviour, screening history, HPV vaccination status, contraception use, smoking and gynaecological history (16–23).

**Figure 1:**
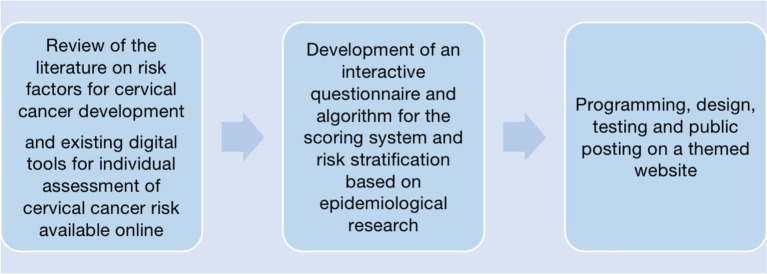
Methodology for development process of the online cervical cancer risk assessment digital tool.

An interdisciplinary development team, including specialists in preventive medicine, public health, epidemiology, and mathematicians/data scientists, translated these risk factors into a structured decision-tree-based logic combined with a cumulative scoring system. The algorithm was developed through an evidence-informed and expert-guided process rather than through statistical modelling of empirical clinical or screening datasets. Accordingly, weight allocation and threshold definitions were determined pragmatically based on published relative risk patterns and expert consensus, rather than derived from calibration against Croatian outcome data (Figure 1).

In the early development phase, existing cervical cancer risk self-assessment tools and online calculators were reviewed (22, 23) and informed the overall structure of the questionnaire, while ensuring adaptation to the Croatian context and national guidelines.

**Figure 2:**
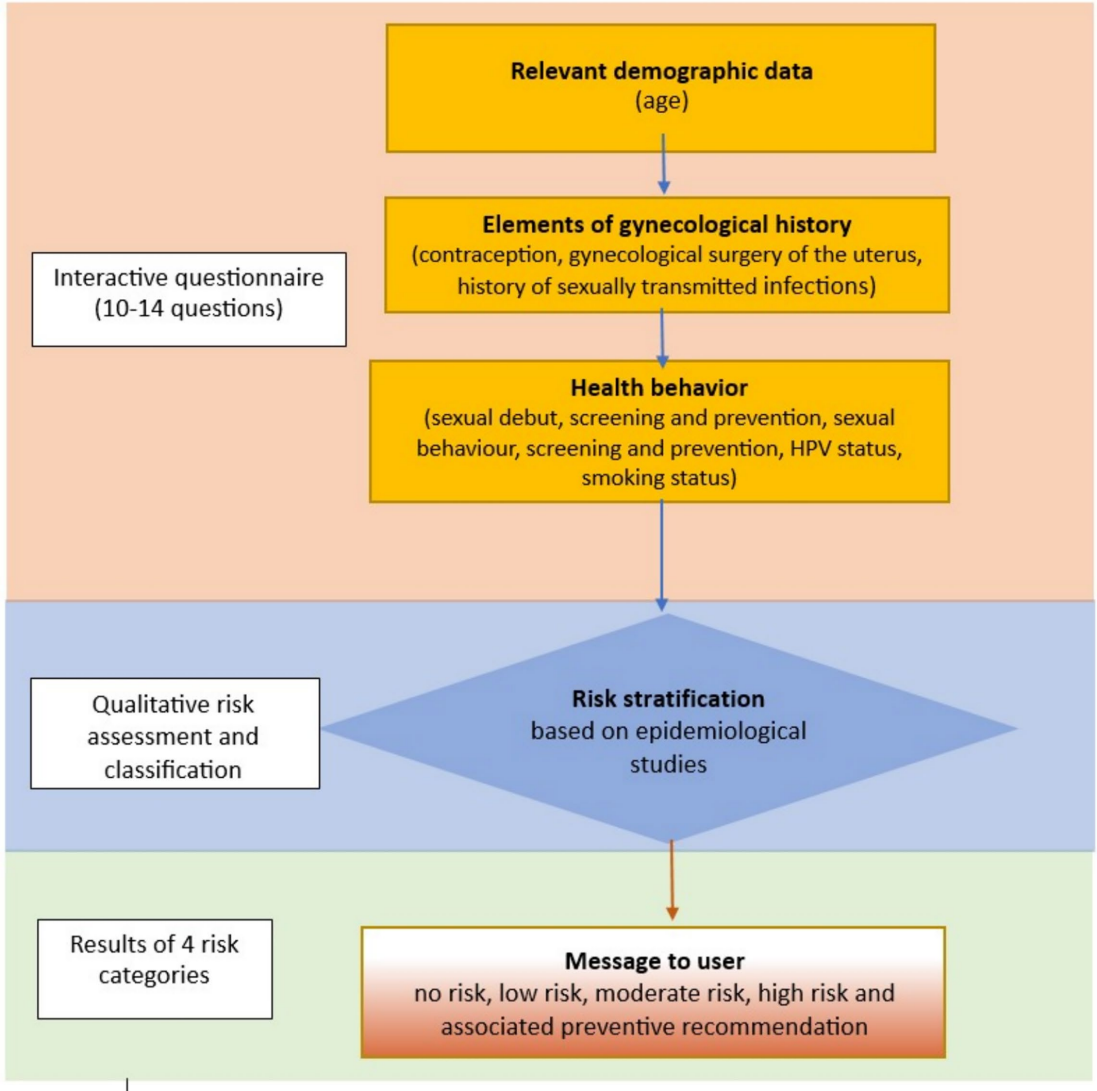
Schematic representation of the questionnaire structure and qualitative risk assessment scoring system.

**Figure 3:**
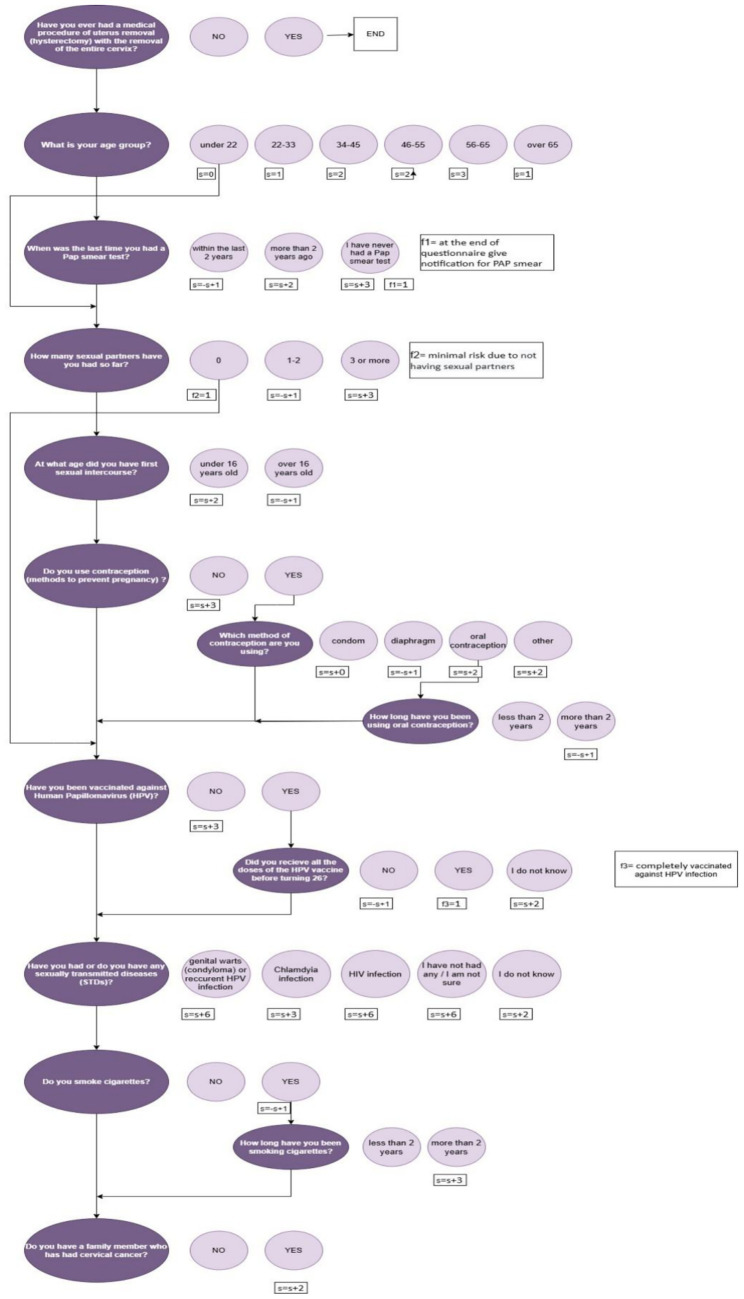
Schematic representation of the development of algorithms for the cervical cancer risk assessment questionnaire.

The decision-tree algorithm uses a combination of (Figures 2, 3):

1. Key binary flags (f₁, f₂, f₃) for variables considered clinically decisive in the categorisation process (e.g., never having a Pap test despite being in the screening age group, active HPV infection, complete hysterectomy with cervix removal).
2. A cumulative score is based on weighted responses, with weights derived from relative risk estimates in the literature (16–23) and expert consensus (Figure 3).

The maximum possible score is 29, allowing for a wide range of stratification within a score-based system. The model classifies users into four qualitative categories: No risk (post-hysterectomy with complete removal of the cervix), Low risk, Moderate risk, and High risk.

In practice, risk categorisation combines thresholds on the total score with the presence or absence of key flags (33). For example, a user over a specified age who reports never having had a Pap test may be classified as high risk regardless of the total score, whereas a user who reports hysterectomy with cervix removal is classified as having no risk of cervical cancer but still receives recommendations for general gynaecological care.

To assess internal logical consistency during development, simulated risk profiles representing diverse combinations of questionnaire responses were constructed. These simulations were used to explore how different response patterns were classified within the rule-based structure and to refine threshold placement in accordance with expert judgement and preventive care priorities. This process was exploratory and did not constitute empirical validation or performance testing against clinical outcome data.

The C2RC algorithm is not a clinically validated prediction model and does not provide an epidemiological risk estimate. It functions as an informational stratification tool designed to structure personalised educational feedback. Given the absence of external validation using population-level outcome data, potential misclassification cannot be excluded, and formal evaluation of performance characteristics remains necessary.

### 3.2 Questionnaire structure and content

The C2RC questionnaire is delivered as a web-based, mobile-responsive interactive form. From the initial concept to the technical solution, development required approximately six months.

Questions are divided into three main categories (Figure 2):

1. Basic demographic data: age (categorised according to screening recommendations and epidemiological risk).
2. Gynaecological history and reproductive health profile: history of hysterectomy and type of surgery (with or without cervix removal), use and duration of hormonal contraception, history of sexually transmitted infections, including HPV, history of gynaecological surgery of the uterus.
3. Health behaviours and preventive practices: age at sexual debut, number of sexual partners, screening history (Pap test and/or HPV testing) and regularity, HPV vaccination status and number of doses, smoking status and duration of smoking, use of gynaecological health services.

The core questionnaire comprises 10 questions, with up to four sub-questions shown through conditional branching. One to two sub-questions appear in three items—for example: number of HPV vaccine doses (if vaccinated), type of contraception and duration of hormonal contraception use, number of cigarettes smoked and duration of smoking (for smokers).

Sub-questions are displayed only when relevant, based on previous answers. This conditional logic ensures that only pertinent questions are shown, thereby reducing respondent burden and improving the precision of risk assessment (Figure 3).

For educational purposes, each question includes links labelled “Why it is important” and “More information,” providing a short explanation of the question’s relevance and a link to authoritative resources on the “Neću rak” platform or other trusted sites. The introduction to the questionnaire explains the purpose of the tool, includes an informed consent text, and emphasises that the tool is intended for education and awareness, not as a substitute for medical consultation or examination.

At the conclusion of the questionnaire, users receive (Table 1):

1. A risk category (qualitative risk assessment 4-level classification: no, low, moderate, high),
2. A brief explanation of what the category means,
3. Tailored recommendations (e.g., schedule a gynaecological examination, undergo Pap or HPV testing, discuss HPV vaccination, or continue current protective behaviours),
4. Reminders of relevant risk and protective factors.

**Table 1:** Categories of risks according to the answers (qualitative risk assessment and classification).

Risk category	High risk	Moderate risk	Low risk	No risk (post-hysterectomy with cervix removal)
Variables that direct defines the risk category or increases or decreases risk (f)*	“f1” - e.g., absence of Pap testing ever in life in someone over age 23 triggers a high-risk classification	“f3” - fully vaccinated against HPV and s ≥ 14	“f2” - has not had any sexual partners OR “f3” - fully vaccinated against HPV and s < 14	No relevant
Score – based stratification (s)** In cases not captured by the above criteria, individuals are categorised based on their total score s	≥ 16	8 ≤ s < 16	< 8	No relevant
Recommendations	Individuals classified as high risk receive a prompt to contact their gynaecologist immediately to discuss the need for a screening test, such as a Pap test or HPV test. This guidance prioritizes early detection, recognising the heightened likelihood of cervical abnormalities within this group.	For those identified with a moderate risk, the recommendation is to schedule a gynaecological visit in the near future. This ensures timely monitoring and the opportunity for further risk assessment if needed.	Individuals in the low-risk category are encouraged to continue protective behaviours and maintain regular gynaecological exams. This recommendation supports preventive care through consistent monitoring and healthy practices.	If the questionnaire indicates that a participant has had a hysterectomy with complete removal of the cervix (first question), the result message informs user of the absence of cervical cancer risk. However, they are still advised to engage in protective behaviours and attend regular gynaecological check-ups for the prevention of other health conditions.

The system uses specific conditions (indicated as variables like “f1,” “f2,” “f3”) and an overall score, “s” to flag individuals into one of three risk categories.

^*^Key variables (flags) “f1,” “f2,” and “f3” directly determine the risk category or increase or decrease the risk and categorise the risk level, regardless of the number of “s” points. The combinations include risk categorization based solely on the score range, direct variables regardless of the number of points, and a combination of points and key variables.

^**^The maximum possible score is 29, allowing for a wide range of stratification within a score-based system. By combining both specific flags and a continuous scoring system, this tool aims to balance the sensitivity and specificity required for a public health-oriented cervical cancer risk stratification model. This scoring approach allows for adjustments depending on resource availability and target population characteristics, providing a flexible yet effective strategy for early identification and intervention among individuals at risk for cervical cancer. The scoring system was designed to be conservative, with the goal of capturing approximately 85% of high-risk individuals while keeping the over-classification rate (i.e., individuals classified too strictly) below 20%. This conservative approach prioritizes public health safety, ensuring a low threshold for further screening recommendations while minimising unnecessary alarm. However, the interactive questionnaire system allows for redefining risk levels in terms of a more lenient or stricter classification. For example, to refine specificity, alternative thresholds can be used for score-based categorization as follows: s < 6: Low Risk; 6 ≤ s < 11: Moderate Risk; s ≥ 11: High Risk. These stricter criteria reduce the threshold for moderate and high risk, providing an additional layer of caution, especially useful in settings with limited resources where higher specificity in high-risk classification can optimise healthcare utilisation.

### 3.3 Programming, implementation, and data protection

The software solution and design of the online interactive questionnaire and algorithm were developed by IT experts in collaboration with the public health team. The tool was implemented on the existing “Neću rak” website of the Croatian Institute of Public Health.

To address the purpose of the digital tool, ethical issues, and data protection, specific Terms and Conditions of Use and an Informed Consent statement were developed for the C2RC. The system is designed to protect privacy so that no personal identifiers (e.g., name, contact details, IP address) are stored, and only aggregated data on users’ answers are collected. Standard web analytics tools (e.g., Google Analytics) are used to capture de-identified statistics such as page views and session duration.

Members of the development team tested the questionnaire extensively prior to public release, both to verify technical performance and to evaluate clarity and usability from an end-user perspective. Minor adjustments were made based on this internal testing.

### 3.4 Usage monitoring and data usage analysis

This community case study analyses aggregated usage data from the period February 2023 to February 2025. Two complementary data sources were used:

1. Web analytics (Google Analytics): number of page views for the informational page and the interactive questionnaire, number of users accessing the page, average session duration, relative contribution of the C2RC to overall “Neću rak” website traffic, temporal distribution of visits, including peaks during campaign periods.
2. Internal questionnaire usage statistics: number of questionnaire starts and completions, age distribution of respondents, aggregated distribution of key responses (e.g., HPV vaccination status, HPV infection history, smoking status, screening history), distribution of risk categories assigned by the algorithm.

Descriptive statistics were used to summarise these data. No inferential analyses were conducted, as the primary goal was to describe reach, user characteristics and early usage patterns. Because data were fully anonymised and aggregated at the level of response categories, individual-level linkage to clinical records or outcomes was not possible.

#### 3.4.1 Reach and user characteristics

From its launch in February 2023, until February 2025, the C2RC content page was viewed almost 9,000 times, contributing approximately 5–10% of total searches on the “Neću rak” website over this period. Around 60% of users who visited the informative page proceeded to the interactive questionnaire. The average time spent on the page was slightly over one minute, suggesting that visitors engaged with the content and, in many cases, completed the questionnaire and reviewed the results. Internal usage statistics show that the interactive questionnaire was completed almost 8,000 times during the observation period. Most respondents (about 60%) were aged 34–54 years, a key target group in which cervical cancer risk increases and organised screening is strongly recommended. The proportion of younger and older users was smaller but non-negligible, indicating some reach beyond the core target age range.

#### 3.4.2 Descriptive questionnaire response patterns

Aggregated questionnaire response statistics derived from routine usage logs of the C2RC describe patterns among completed questionnaires rather than behavioural characteristics of a defined population. Among completed questionnaires, 13% included a self-reported history of HPV vaccination, and 13% indicated a previous HPV infection. Approximately 30% of completed questionnaires reported cigarette smoking; among these responses, around 95% indicated smoking for more than two years.

The algorithm’s risk classification output (no, low, moderate, high risk) reflects these patterns in combination with age, screening history, sexual behaviour, and other factors. Although detailed distribution by risk category is not presented here, preliminary internal analyses suggest that a non-trivial share of users fall into moderate and high-risk categories, often driven by under-screening and lack of HPV vaccination. However, these distributions reflect tool-generated outputs rather than validated epidemiological risk estimates.

#### 3.4.3 Temporal patterns and linkage with campaigns

Analysis of temporal patterns in web analytics shows that visits to the C2RC page and completions of the questionnaire were consistently observed throughout the year, with two clear peaks during January and February of both 2024 and 2025. These peaks coincided with European Cancer Prevention Week, and National Cervical Cancer Prevention Day (Mimosa Day), accompanied by intensified online campaigns via the “Neću rak” website and social media accounts of the Croatian Institute of Public Health. This pattern suggests that integrating the tool into broader awareness campaigns can substantially amplify its reach.

## 4 Discussion

### 4.1 Summary of the case

This community case study describes the development and early implementation of a national, web-based cervical cancer risk calculator integrated into the Croatian Institute of Public Health’s “Neću rak” platform. The tool combines an epidemiologically grounded decision-tree algorithm, an adaptive questionnaire and embedded educational content. During its first two years online, the tool was accessed almost 9,000 times, with approximately 8,000 completed questionnaires. Most users were women in the key screening age group, yet HPV vaccination and non-smoking behaviours were far from universal, underscoring persistent prevention gaps.

### 4.2 Contribution to digital health literacy and prevention

The C2RC is consistent with WHO’s classification of digital health interventions for individuals—specifically, tools initiated by users that provide health information and support decision-making via websites or applications (6, 7, 24). By combining self-assessment with tailored educational messages and “Why it is important” explanations for each question, the tool aims to draw attention to both functional health literacy (understanding of basic concepts) and interactive health literacy (ability to relate information to personal circumstances) in the target population.

Prior research suggests that digital interventions—including web tools, mobile applications and social media campaigns—can enhance awareness and support adherence to screening and vaccination programmes (25–27). The observed usage patterns in this case study indicate that a national public health institution can leverage a relatively simple digital tool to engage a substantial number of women, particularly those in the high-priority age range for cervical cancer screening. The direct and immediate feedback (risk category plus recommendations) may also help transform abstract prevention messages into more personally meaningful guidance, or at least spark interest in given topics.

### 4.3 Positioning within existing digital tools

Several digital tools have been developed internationally to support cervical cancer prevention, including online risk calculators, web-based decision aids, and mobile apps that address HPV-related concerns or provide counselling after screening (22, 23, 28–30, 34, 35). Recent meta-analytic evidence indicates that digital education interventions are associated with improvements in HPV-related knowledge and vaccination engagement among adolescents and young adults (31), situating such tools within a broader ecosystem of digital prevention strategies.

Within this broader landscape, C2RC should be understood as a national-level adaptation embedded within the Croatian public health infrastructure rather than as a fundamentally distinct innovation. Similar to other digital risk assessment tools, it combines structured self-reported inputs with rule-based categorisation. However, its implementation within the official “Neću rak” platform allows alignment with national screening and vaccination pathways and direct linkage to publicly endorsed educational resources.

In addition, personalised feedback incorporates educational materials, including a short film developed within the EU Interreg project “Before Time” (32). This integration connects self-assessment outputs with structured informational content and reflects ongoing European initiatives aimed at strengthening cervical cancer prevention through coordinated communication strategies.

## 5 Lessons learned

Several practical lessons emerged from the development and early implementation of the C2RC:

*Lesson 1* – Hosting within a trusted public health platform facilitates uptake.

Placing the tool on the “Neću rak” website of the Croatian Institute of Public Health, rather than as a stand-alone app, likely contributed to sustained traffic and engagement. The association with a recognised public institution may reduce scepticism and encourage use among women who might otherwise be cautious about online health tools.

*Lesson 2* – Conditional logic supports brevity and relevance.

By using conditional branching, the questionnaire maintains a manageable length (10–14 questions) while capturing key risk factors in greater detail when needed. Users encounter only questions relevant to them, which likely contributes to the observed completion rates and short but meaningful session durations.

*Lesson 3* – Conservative risk thresholds are acceptable in a public health education tool.

Designing the algorithm to capture approximately 85% of high-risk individuals, at the cost of some over-classification, was considered appropriate for a national awareness tool. This “pessimistic” approach minimises the risk of underestimating risk in high-need individuals, as long as the communication clearly frames the tool as educational and encourages users to discuss results with healthcare providers.

*Lesson 4* – Alignment with campaigns amplifies impact.

Timing promotional efforts around European Cancer Prevention Week and Mimosa Day substantially increased usage, showing that digital tools can effectively complement broader awareness campaigns when integrated into their communication plans.

*Lesson 5* – Interdisciplinary collaboration is essential.

The collaboration between preventive medicine specialists, public health experts, mathematicians/data scientists and IT professionals was critical to balancing epidemiological rigor, user-friendly design and technical robustness.

## 6 Conceptual and methodological constraints

The current usage analysis relies on anonymised, aggregated web and questionnaire data which are routinely captured through the interactive questionnaire in the administrative interface. It is not possible to determine whether using the C2RC led to increased HPV vaccination, more regular Pap or HPV testing, or earlier detection of cervical lesions or cancer. We did not assess users’ satisfaction, usability experience, or changes in knowledge and attitudes after using the tool. Therefore, no conclusions can be drawn regarding effects on health literacy or behavioural outcomes. The decision-tree model was initially calibrated using synthetic patient profiles. While this approach allowed rapid iteration, it may not fully capture the complexity of real-world populations. The interactive questionnaire in C2RC has not been externally validated, and drift and calibration issues are beyond this study’s scope. Furthermore, the scoring framework does not provide a clinically validated prediction of cervical cancer and should not be interpreted as an epidemiological risk estimate.

The conservative thresholds may lead to overestimation of risk in some users, which is acceptable for an awareness tool but may reduce perceived specificity. Users of the tool represent a self-selected subgroup with probably higher digital literacy, greater motivation for preventive behaviours, and potentially higher baseline health awareness than the general female population. As a result, women with limited internet access, lower health literacy, or structural barriers to healthcare are likely to be under-represented, which limits the generalizability of the findings and constrains the overall public health impact of the digital tool. Although the questionnaire and educational texts were written in accessible language, we did not formally assess readability levels or adapt content for different literacy groups or languages beyond Croatian. Future versions could incorporate simplification, multiple language options and multimedia formats.

Addressing these constraints will be important in future research phases, which may include mixed-methods evaluation, user surveys and, where ethically and legally feasible, linkage to screening and vaccination data.

## 7 Conclusion

Education and awareness about risk factors, protective behaviours, and the importance of screening and vaccination are central components of cervical cancer prevention. In this community case study, the integration of an evidence-informed scoring framework with a digital platform illustrates the feasibility of delivering structured prevention-related information within a national public health setting. The tool enables users to receive personalised educational feedback aligned with existing screening and vaccination pathways.

As a digital self-assessment instrument, C2RC represents one possible informational resource within broader health promotion strategies rather than a substitute for clinical risk assessment or organised screening programmes. Its potential role lies in supporting communication and access to standardised information. Formal evaluation of algorithm performance, user experience, and possible impacts on knowledge or behaviour is required before drawing conclusions regarding effectiveness.

## Data Availability

The raw data supporting the conclusions of this article are available from the authors upon reasonable request.
